# Severe Acquired Hypothyroidism and Van Wyk–Grumbach Syndrome in Two Children

**DOI:** 10.1155/2024/8919177

**Published:** 2024-07-09

**Authors:** Corina Ramona Nicolescu, Lucie Bazus, Jean-Louis Stephan

**Affiliations:** ^1^ Department of Pediatric Endocrinology and Diabetes Centre Hospitalier Universitaire Saint-Etienne, Avenue Albert Raimond, Saint-Priest en Jarez 42270, France; ^2^ Department of Pediatrics Centre Hospitalier Universitaire Saint-Etienne, Avenue Albert Raimond, Saint-Priest en Jarez 42270, France

## Abstract

The primary manifestations of chronic hypothyroidism in children include growth arrest, delayed skeletal maturity, and delayed puberty. In 1960, Van Wyk and Grumbach reported three girls with hypothyroidism and a combination of incomplete isosexual precocious puberty (early breast development, menstruation, and absence of pubic hair), galactorrhea, delayed bone age, and pituitary enlargement. All abnormalities regressed after appropriate thyroid hormone replacement therapy. Over the years, an increasing number of reported cases has allowed for a more precise understanding of the clinical, biochemical, and radiological phenotypes of the Van Wyk–Grumbach syndrome (VWGS). These varying clinical manifestations are thought to result from a unique pathophysiological process where the thyroid-stimulating hormone (TSH) is a key element. We describe the cases of two patients (a boy and a girl) with severe autoimmune thyroiditis and VWGS. The clinical, biochemical, and radiological imaging characteristics were similar in both patients and included growth failure, absence of clinical goiter, markedly elevated TSH concentrations >100 mIU/L, undetectable free thyroxine levels, “normal” thyroglobulin levels, high follicle-stimulating hormone (FSH) and prolactin levels, prepubertal levels of luteinizing hormone (LH), delayed bone age, and hyperplasia of the pituitary gland. The two patients displayed differences, especially in the absence of clinical pubertal development, moderate anemia, abnormal renal function, and moderate goiter detected via ultrasonography (in the female patient). Thyroxine replacement therapy reversed the VWGS phenotype and hypothyroidism, with satisfactory growth velocity, strictly normal thyroid function, and normal pituitary size detected via magnetic resonance imaging at the 6-month follow-up visit.

## 1. Introduction

Autoimmune thyroiditis (AIT) is the most common childhood thyroid disease. There are two types of AIT: goitrous (Hashimoto's thyroiditis) and nongoitrous (atrophic thyroiditis). AIT prevalence ranges between 1 and 3% and occurs more frequently among girls [1], where it can coexist with other organ-specific autoimmune disorders and chromosomal disorders (Down, Turner, and Klinefelter syndromes).

The destruction and progressive failure of the thyroid gland is a complex immune-mediated process involving cell-mediated and humoral mechanisms and the secretion of antibodies (Abs) against a variety of thyroid-specific antigens, including thyroglobulin (Tg), thyroid peroxidase (TPO), thyroid-stimulating hormone receptor (TSHR), sodium iodide symporter (NIS), and pendrin [2].

The clinical presentation of AIT is insidious, and symptoms and signs are heterogeneous. In children with severe or long-lasting disease, linear growth retardation and developmental/pubertal delays are the most informative features. Severe hypothyroidism is rarely associated with precocious puberty, as observed in the VWGS.

Originally described in 1960 as a combination of hypothyroidism and precocious puberty (with delayed bone age and ovarian cysts in girls) [3], VWGS is now defined by specific clinical, biological, and radiological profiles. The clinical phenotype includes signs of severe hypothyroidism and incomplete isosexual precocious pubertal development (without virilization) in both sexes. Biological findings include extremely elevated TSH levels with low/undetectable free thyroxine (fT4) levels, elevated FSH and estradiol levels, suppressed LH levels, and very high prolactin levels. The radiological findings include delayed skeletal maturation (bone age), bilaterally enlarged ovaries with multiple follicular cysts, and an enlarged pituitary gland.

The pathogenesis of VWGS is explained by a complex mechanism of tight interactions/crosstalk between hypothalamic-pituitary hormones, with overlapping actions at the receptor level.

Thyroid hormone deficiency induces excessive pituitary TSH secretion by a negative feedback mechanism and the resulting high TSH levels act as an “FSH-like” agonist on the FSH receptors in the ovary and testis because TSH and FSH share a commonality in their *α*-subunits.

Hyperprolactinemia can be explained by (a) pituitary stalk compression due to pituitary thyrotrophic hyperplasia, disruption of hypothalamic inhibition, and/or (b) thyrotropin-releasing hormone (TRH)-related hyperprolactinemia with a progressive reduction in hypothalamic gonadotropin-releasing hormone (GnRH) pulse frequency.

Treatment of both hypothyroidism and VWGS involves hormone replacement with correction of clinical and biological hypothyroidism, regression of precocious pubertal signs, amelioration of pituitary hyperplasia, and favorable long-term outcomes.

We present two cases of children with severe primary hypothyroidism and VWGS syndrome. They were treated with thyroxine replacement therapy with favorable clinical, biological, and radiological outcomes.

## 2. Case 1

A 10-year-old boy with minimal linear growth between the ages of 7 and 10 years was referred to the endocrinology department. His parents reported his growth arrest over the last two years (growth velocity: 0 cm/24 months), worsening lethargy for several months, and constipation. He also complained of difficulties at school, worsening school performance, and extreme fatigue. No neurological (headaches) or visual symptoms were observed. The medical history and family history of the patient were unremarkable.

Upon general physical examination, his anthropometric parameters were as follows: weight 31 kg (25^th^ percentile), height 126 cm (<3^rd^ percentile), and normal vital signs, except for a heart rate (HR) of 66 beats/min. He was cooperative, but lethargic, with slowed mentation. He appeared pale, with periorbital puffiness, brittle hair, and dry skin. The intestinal transit was delayed (constipation). He had no goiter. His genitalia had an atypical pubertal appearance, with bilaterally enlarged testes (8 mL), stretched penile length of 3.8 cm, and a lack of pubic hair. Neurological and visual field examination results were normal.

The results of tests for complete blood count, electrolyte, glucose, liver, and renal function were normal. His total cholesterol and triglyceride levels were elevated.

Primary hypothyroidism was diagnosed on the basis of a TSH concentration of 2400 mIU/L, with an fT4 concentration of 1 pmol/L, and a thyroglobulin level of 21 *μ*g/L. AIT was confirmed based on elevated thyroid antibody levels (Table 1).

Other notable hormonal abnormalities observed in this patient included elevated prolactin and FSH levels, and low LH levels (elevated FSH/LH ratio) (Table 1). Adrenal (cortisol) and somatotroph (insulin-like growth factor 1 (IGF1) and insulin-like growth factor binding protein-3 (IGFBP3)) functions were normal.

Thyroid ultrasonography (US) revealed a normal thyroid gland with homogenous mild hypoechoic changes and color Doppler showed diffuse hypervascularity of the hypoechoic lesions. His bone age was 6 years (according to the Greulich and Pyle Atlas).

**Figure 1:**
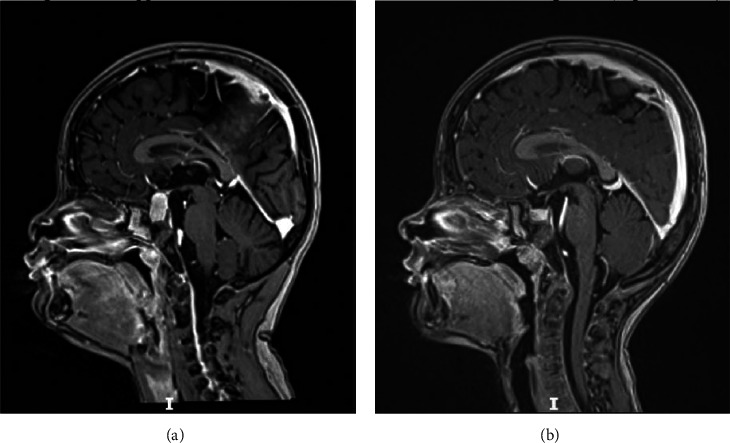
Magnetic resonance imaging (MRI) sagittal T1-weighted image sequences. (a) Pituitary hyperplasia (diameters 18 × 12 × 17 mm, convex upper margin). (b) Regression of pituitary dimensions 6 months after presentation.

Magnetic resonance imaging (MRI) of the hypothalamus and pituitary gland revealed an enlarged, homogenous pituitary gland with a craniocaudal diameter of 18 mm and a convex superior margin. The posterior pituitary showed a high signal intensity. The optic chiasm was not affected (Figure 1(a)).

A diagnosis of atrophic AIT and VWGS was made, and hormone replacement therapy with levothyroxine (5 *μ*g/kg/d) was initiated.

At the 6-month follow-up, the patient showed resolution of symptoms, favorable catch-up growth (5 cm/6 months), a loss of 3 kg, and unchanged testicular enlargement. The thyroid function was normal (Table 1), and the pituitary dimensions regressed (pituitary height 9 mm) with a homogenous appearance on the second MRI images (Figure 1(b))

## 3. Case 2

A previously healthy 6-year-old girl was referred to the endocrinology department for further investigation of a 24-month history of growth arrest, fatigue, constipation, and cold intolerance. The patient showed no neurological symptoms. The medical history and family history of the patient were unremarkable.

On physical examination, her vital signs were normal; however, she had bradycardia (HR 64 beats/min). Her body weight was 18 kg (50^th^ percentile) and her height was 102 cm (<3^rd^ percentile). She appeared pale with generalized puffiness (face and extremities) and dry skin. Her thyroid gland was not clinically enlarged, and she had Tanner stage 1. Deep tendon reflexes exhibited a slowed relaxation phase. Her neurocognitive development was normal.

She had mild normocytic normochromic anemia (hemoglobin level 8.4 g/dL), moderately impaired renal function (creatinine level 63 *μ*mol/L), and dyslipidemia (Table 1).

A severe primary hypothyroidism of autoimmune etiology was revealed by an undetectable fT4 level of 1.1 pmol/L with a TSH level of 2200 mIU/L, and the presence of anti-TPO and anti-Tg antibodies.

Other hormonal abnormalities included prolactin (2127 mIU/L), FSH (6.6 IU/L), estradiol (12 ng/L), and undetectable LH levels (high FSH/LH ratio). Her serum cortisol, IGF_1_, and IGFBP_3_ levels were normal.

Ultrasonography of the thyroid gland showed a moderate goiter with a markedly heterogeneous echo texture, hypoechoic areas, and hypervascularization of the gland on color Doppler imaging. Her bone age was delayed by 3 years compared with her chronological age. Pelvic ultrasonography showed that her uterus and ovaries were normal for her age (uterus 32 mm in length with normal endometrium and normal-sized ovaries without follicular cysts).

**Figure 2:**
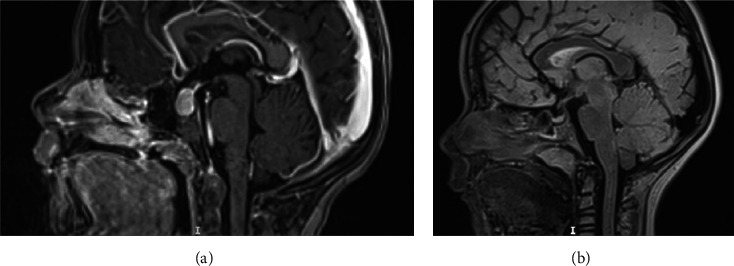
Magnetic resonance imaging (MRI) sagittal T1-weighted image sequences. (a) Pretreatment pituitary hyperplasia (craniocaudal diameter 14 mm, convex upper margin, and no contact with the optic chiasm). (b) Posttreatment normal pituitary diameter (4 mm).

Pituitary MRI showed hyperplasia of the anterior gland (craniocaudal diameter 14 mm) with homogenous enhancement and an unremarkable posterior pituitary (Figure 2(a)).

A diagnosis of severe acquired hypothyroidism (Hashimoto's thyroiditis) and VWGS was made and treatment with L-T4 hormone was started (dosage 5 *μ*g/kg/d).

Six months after the presentation, the patient was doing well, had gained 7.5 cm in height, had lost 2 kg, and displayed a euthyroid state. Dynamic MRI showed normalization of the pituitary size (Figure 2(b)).

## 4. Discussion

The two patients described in this case report presented with the shared clinical, biological, and radiological features of long-standing acquired hypothyroidism and VWGS, although some differences were observed.

The female patient presented with moderate normocytic anemia. Anemia is a common finding in patients with hypothyroidism [4, 5] and different types have been described (normochromic normocytic, hypochromic microcytic, and megaloblastic) [6, 7] that have been explained by varying mechanisms including thyroid hormone deficiency (lack of stimulation of erythroid precursors) in normocytic anemia, menorrhagia and iron malabsorption in microcytic anemia, and malabsorption of vitamin B12 and folic acid in the macrocytic form.

A detailed hematological assessment of anemia in children with hypothyroidism is essential because it is a treatable condition where the treatment depends on the cause.

With regard to the moderately altered renal function in the female patient, the interplay between thyroid and renal function has been well described in the literature [8, 9]. Thyroid hormones influence the development, structure, and function of the kidneys. Hypothyroidism causes renal dysfunction directly via glomerular and tubular functions (increased serum creatinine and decreased glomerular filtration rate), and indirectly via prerenal function (cardiovascular hemodynamics and renal blood flow) [10–12]. Renal function impairment is transient and reversible following thyroxine replacement therapy [11].

In patients with primary profound hypothyroidism, growth hormone production may be altered [13]. In our patients, the pituitary profile revealed normal growth hormone secretion (IGF_1_ and IGFBP_3_). Treatment with levothyroxine promoted a rapid increase in growth velocity.

As in the majority of children with VWGS syndrome, our patients had elevated prolactin, moderately elevated FSH, and prepubertal LH levels. However, the clinical expression of this particular gonadotropic profile differed between the two patients. The male patient presented with clinical features of FSH-mediated sexual changes (isolated testicular enlargement), whereas the female patient lacked these features (absence of breast development, enlarged ovaries with follicular cysts, and menstruation).

Incomplete isosexual pubertal precocity in children with VWGS is characterized by a unique aggregation of retarded linear growth, pubertal development without virilization, elevated FSH levels with prepubertal LH levels, high prolactin levels, and delayed bone age.

Boys with VWGS display macroorchidism without pubic or axillary hair, and their testicular histology shows a predominance of tubular elements without increased Leydig cell numbers, consistent with an FSH-mediated response [14, 15].

Girls with VWGS may exhibit varying degrees of pubertal development, including breast development with or without galactorrhea, uterine bleeding before the onset of pubic or axillary hair growth, and bilaterally enlarged/multicystic ovaries (sometimes giant ovaries). Hypothyroidism-mediated ovarian stimulation can be severe, with a presentation indicating a surgery emergency (ovarian torsion) [16, 17]. Our female patient showed no clinical signs of puberty or enlarged ovaries as detected via the US.

Several hypotheses involving pathophysiological mechanisms have been proposed to explain hypothyroidism-induced precocious puberty.

Van Wyk and Grumbach proposed a pituitary hormonal overlap underlying the role of elevated TSH, FSH, and prolactin in pubertal activation. The pituitary gland secretes large quantities of TSH, becomes hyperplastic, and secretes gonadotropins and prolactin [3]. The currently debated mechanism, “specificity spillover,” states that high levels of TSH activate FSH receptors because of the molecular similarities between the two glycoprotein pituitary hormones (a common *α*-subunit and a uniquely specific *β*-subunit). TSH acts as a gonadotropin in the ovaries or testes of patients with VWGS and its effects are determined not by the hormone but by the receptor that is activated. Very high TSH concentrations can directly activate the wild-type FSH receptor in the absence of mutations in the human FSH receptor [18, 19].

What is the role of prolactin in gonadal stimulation during VWGS? Hyperprolactinemia is a common feature in children with this syndrome and is TRH-mediated. Increased TRH production stimulates both thyrotrophic and lactotrophic pituitary cells, resulting in pituitary enlargement and hyperprolactinemia (due to the cross-sensitivity between TSH and prolactin-producing pituitary cells to TRH stimulation) [20]. Estrogens may also be involved in this process [21].

Hyperprolactinemia suppresses the pituitary-gonadotropin axis by slowing the pulse frequency of GnRH [22–24] with FSH-predominant gonadotropin secretion and simultaneous suppression of LH [25, 26], and this differential regulation (dependent on prolactin levels) may explain the discordance between FSH and LH in this syndrome. In addition, hyperprolactinemia increases ovarian sensitivity to circulating FSH [27], further explaining the US imaging features of follicular maturation and multicystic changes found in girls with VWGS.

Pituitary hyperplasia due to severe primary hypothyroidism was first described by Niepce in 1851 [28]. It refers to the diffuse enlargement of the anterior pituitary lobe caused by the loss of negative feedback, with increased hypothalamic TRH secretion accompanied by thyrotrophic, and sometimes lactotrophic hyperplasia.

There is a high incidence of pituitary hyperplasia in adult patients with hypothyroidism, with a significantly positive correlation between the size of the enlarged pituitary and TSH levels [29].

There is a paucity of information regarding the pediatric population, and pituitary hyperplasia remains under-recognized in this cohort. It is mostly asymptomatic in children (while exhibiting neurological and visual complications in adult patients) and has a characteristic MRI appearance (homogenous, symmetrically enlarged pituitary with a convex upper border, suprasellar extension, and variable compression of the optic chiasm). Clinical, biological, and radiological follow-ups help to differentiate between adenomas and other primary pituitary lesions.

In all reported pediatric cases, regression of pituitary hyperplasia and correction of the clinical and biochemical abnormalities of hypothyroidism have been noted.

The absence of goiter is another common feature in patients with severe hypothyroidism. The two patients described here exhibited normal thyroglobulin levels that were inappropriately low for their very high TSH levels, and an absence of clinical goiter, although a moderately enlarged thyroid was observed in the female patient.

In AIT, the mechanisms of thyroid gland cell death vary among patients, and the primary causes of decreased thyroid hormone production are immune-mediated apoptosis and cytolysis of thyrocytes. Another mechanism involves TSHR-blocking antibodies that prevent TSH from stimulating thyrocytes and inhibit TSH-induced cell proliferation and hormone synthesis [30].

In adults, TSHR-blocking antibodies appear to be more common in patients with nongoitrous AIT than in those with the goitrous form [31], although one of the initially reported TSHR antibody-positive patients had severe goitrous hypothyroidism [32].

In the pediatric population, a recent study [33] reported that TSHR-blocking antibodies can be found in patients with either a goiter or an atrophic gland, although TSHR antibody-positive children were significantly more likely to be hypothyroid and not have a goiter at the time of diagnosis than TSHR antibody-negative patients.

High titers of TSHR-blocking antibodies can persist and cross the placenta in adolescents and young women with a risk of fetal/transient neonatal hypothyroidism [34–37].

TSHR-blocking antibodies were not tested in our patients.

## 5. Conclusion

VWGS is common in children with severe hypothyroidism and should be considered when a patient presents with growth arrest, incomplete precocious puberty, delayed bone age, and pituitary hyperplasia. Understanding its pathophysiology, mediated by markedly elevated TSH levels, can help guide the appropriate diagnostic approach and initiate levothyroxine treatment with the restoration of a euthyroid state.

Hypothyroidism is not a condition indicating surgery; however, VWGS may lead to several clinical emergencies (abdominal due to ovarian torsion, and neurological or visual due to pituitary hyperplasia) where surgical interventions (oophorectomy or pituitary biopsy) can be appropriately avoided. Ovarian hyperstimulation with bilaterally cystic giant ovaries and pituitary gland enlargement deserve a multidisciplinary approach, with a special emphasis on growth patterns and thyroid function.

## Figures and Tables

**Table 1:** Diagnostic and follow-up findings in the patients.

Parameters (reference range)	Patient 1		Patient 2	
	At diagnosis	6 months later	At diagnosis	6 months later
Clinical				
Weight	31 kg (25^th^ P)	27 kg	18 kg (25^th^ P)	18 kg
Height	126 cm (<3^rd^ P)	131 cm (+5 cm/6 months)	102 cm (<3^rd^ P)	109.5 cm (+7.5 cm/6 months)
Thyroid	No goiter		No goiter	
Pubertal development	Testicles of 8 mL	Testicles of 8 mL	Tanner stage 1	
Biological				
Hemoglobin (11–14.7 g/dL)	12		8.4	11.2
Nitrogen urea (2.6–6 mmol/L)	5		5.7	
Creatinine (27–53 *μ*mol/L)	52		63	36
Total cholesterol (4.3–5.2 mmol/L)	6.7		6	
Triglycerides (<1.70 mmol/L)	3.2		2.45	
fT4 (12.5–21.5 pmol/L)	1	18.6	1.1	20
TSH (0.6–4.8 mIU/L)	2400	66	2200	0.63
Thyroglobulin (3.5–77 *μ*g/L)	21		63	
Anti-Tg Ab (<40 IU/mL)	110		537	
Anti-TPO Ab (<25 IU/mL)	508		1236	
FSH (0.1–4.3 IU/L)	6.4		6.6	
LH (0.1–5 IU/L)	<0.3		<0.3	
Prolactin (100–496 mIU/L)	2045	342	2127	287
Estradiol (6–27 ng/L)			12	
Radiological				
Thyroid ultrasound	Normally sized gland with alterations of echogenicity and homogeneity of the parenchyma		Moderate goiter with heterogeneous structure	
Bone age	6 years		3 years	
Pituitary MRI	18 × 12 × 17 mm	9 × 9 × 9 mm	14 × 10 × 10 mm	4 mm
Pelvic US			Prepubertal uterus and ovaries	
Treatment				
L-T4	5 *μ*g/kg/d	2 *μ*g/kg/d	5 *μ*g/kg/d	2.2 *μ*g/kg/d

P, percentile; fT4, free thyroxine; TSH, thyroid-stimulating hormone; Tg, thyroglobulin; TPO, thyroid peroxidase; Abs, antibodies; fT4, free thyroxine; FSH, follicle-stimulating hormone; LH, luteinizing hormone; MRI, magnetic resonance imaging; US, ultrasonography; L-T4, levothyroxine.

## Data Availability

The data used to support the findings of this study are available from the corresponding author upon request.

## References

[1] Kaloumenou I., Mastorakos G., Alevizaki M. (2008). Thyroid autoimmunity in schoolchildren in an area with long-standing iodine sufficiency: correlation with gender, pubertal stage, and maternal thyroid autoimmunity. *Thyroid*.

[2] Brown R. S. (2013). Autoimmune thyroiditis in childhood. *Journal of Clinical Research in Pediatric Endocrinology*.

[3] Van Wyk J. J., Grumbach M. M. (1960). Syndrome of precocious menstruation and galactorrhea in juvenile hypothyroidism syndrome of precocious menstruation and galactorrhea in juvenile hypothyroidism: an example of hormonal overlap in pituitary feedback an example of hormonal overlap in pituitary feedback. *The Journal of Pediatrics*.

[4] Rabet-Bensalah K. M., Aubert C. E., Coslovsky M., Collet T. H., Baumgartner C., den Elzen W. P. (2016). Thyroid dysfunction and anaemia in a large population‐based study. *Clinical Endocrinology*.

[5] Chu J. Y., Monteleone J. A., Peden V. H., Graviss E. R., Vernava A. M. (1981). Anemia in children and adolescents with hypothyroidism. *Clinical Pediatrics*.

[6] Erdogan M., Kösenli A., Ganidagli S., Kulaksizoglu M. (2012). Characteristics of anemia in subclinical and overt hypothyroid patients. *Endocrine Journal*.

[7] Das C., Sahana P. K., Sengupta N., Giri D., Roy M., Mukhopadhyay P. (2012). Etiology of anemia in primary hypothyroid subjects in a tertiary care center in Eastern India. *Indian Journal of Endocrinology and Metabolism*.

[8] Elgadi A., Verbovszki P., Marcus C., Berg U. B. (2008). Long-term effects of primary hypothyroidism on renal function in children. *The Journal of Pediatrics*.

[9] Al-Fifi S., Girardin C., Sharma A., Rodd C. (1999). Moderate renal failure in association with prolonged acquired hypothyroidism in children. *Acta Paediatrica*.

[10] Narasaki Y., Sohn P., Rhee C. M. (2021). The interplay between thyroid dysfunction and kidney disease. *Seminars in Nephrology*.

[11] del-Río Camacho G., Tapia Ceballos L., Picazo Angelín B., Ruiz Moreno J. A., Nieto M. L. H., Romero González J. (2003). Renal failure and acquired hypothyroidism. *Pediatric Nephrology*.

[12] Ellervik C., Mora S., Ridker P. M., Chasman D. I. (2020). Hypothyroidism and kidney function: a mendelian randomization study. *Thyroid*.

[13] Corica D., Abbate T., Kucharska A. M. (2024). Growth impairment in children with atrophic autoimmune thyroiditis and pituitary hyperplasia. *Italian Journal of Pediatrics*.

[14] Anasti J. N., Flack M. R., Froehlich J., Nelson L. M., Nisula B. C. (1995). A potential novel mechanism for precocious puberty in juvenile hypothyroidism. *Journal of Clinical Endocrinology and Metabolism*.

[15] Bruder J. M., Samuels M. H., Bremner W. J., Ridgway E. C., Wierman M. E. (1995). Hypothyroidism-induced macroorchidism: use of a gonadotropin-releasing hormone agonist to understand its mechanism and augment adult stature. *Journal of Clinical Endocrinology and Metabolism*.

[16] Nandi-Munshi A. D., Tridgell A., Taplin C. E. (2013). Acute ovarian torsion and primary hypothyroidism. *Pediatrics*.

[17] Agarwala S., Gupta A. (2018). Primary hypothyroidism presenting as bilateral ovarian torsion. *Indian Pediatrics*.

[18] De Leener A., Montanelli L., Van Durme J. (2006). Presence and absence of follicle-stimulating hormone receptor mutations provide some insights into spontaneous ovarian hyperstimulation syndrome p. *Journal of Clinical Endocrinology and Metabolism*.

[19] Ryan G. L., Feng X., d’Alva C. B. (2007). Evaluating the roles of follicle-stimulating hormone receptor polymorphisms in gonadal hyperstimulation associated with severe juvenile primary hypothyroidism. *Journal of Clinical Endocrinology and Metabolism*.

[20] Durbin K. L., Diaz-Montes T., Loveless M. B. (2011). Van wyk and Grumbach syndrome: an unusual case and review of the literature. *Journal of Pediatric and Adolescent Gynecology*.

[21] Hunold A., Alzen G., Wudy S. A. (2009). Ovarian tumor in a 12‐year old female with severe hypothyroidism: a case of Van Wyk and Grumbach syndrome. *Pediatric Blood and Cancer*.

[22] Denef C. (2008). Paracrinicity: the story of 30 years of cellular pituitary crosstalk. *Journal of Neuroendocrinology*.

[23] Krassas G. E., Poppe K., Glinoer D. (2010). Thyroid function and human reproductive health. *Endocrine Reviews*.

[24] Grattan D. R., Lasoni C. L., Liu X. L., Anderson G. M., Herbison A. E. (2007). Prolactin regulation of gonadotropin-releasing hormone neurons to suppress luteinizing hormone secretion in mice. *Endocrinology*.

[25] Thackray V. G., Mellon P. L., Coss D. (2010). Hormones in synergy: regulation of the pituitary gonadotropin genes. *Molecular and Cellular Endocrinology*.

[26] Clarke S. A., Nesbitt A., Ali S. (2018). Interpretation of serum gonadotropin levels in hyperprolactinaemia. *Neuroendocrinology*.

[27] Advis J. P., Richards J. S., Ojeda S. R. (1981). Hyperprolactinemia-induced precocious puberty: studies on the mechanism(s) by which prolactin enhances ovarian progesterone responsiveness to gonadotropins in prepubertal rats. *Endocrinology*.

[28] Niepce E. B. (1852). Traité du Goître et du Crétinisme. *Bailliere*.

[29] Khawaja N. M., Taher B. M., Barham M. E. (2006). Pituitary enlargement in patients with primary hypothyroidism. *Endocrine Practice*.

[30] Smith B. R., Sanders J., Furmaniak J. (2007). TSH receptor antibodies. *Thyroid*.

[31] Arikawa K., Ichikawa Y., Yoshida T. (1985). Blocking type antithyrotropin receptor antibody in patients with nongoitrous hypothyroidism: its incidence and characteristics of action. *Journal of Clinical Endocrinology and Metabolism*.

[32] Endo K., Kasagi K., Konishi J. (1978). Detection and properties of TSH-binding inhibitor immunoglobulins in patients with Graves’ disease and Hashimoto’s thyroiditis. *Journal of Clinical Endocrinology and Metabolism*.

[33] Feingold S. B., Smith J., Houtz J., Popovsky E., Brown R. D. (2009). Prevalence and functional significance of thyrotropin receptor blocking antibodies in children and adolescents with chronic lymphocytic thyroiditis. *Journal of Clinical Endocrinology and Metabolism*.

[34] Takasu N., Mori T., Koizumi Y., Takeuchi S., Yamada T. (1984). Transient neonatal hypothyroidism due to maternal immunoglobulins that inhibit thyrotropin-binding and post-receptor processes. *Journal of Clinical Endocrinology and Metabolism*.

[35] Brown R. S., Keating P., Mitchell E. (1990). Maternal thyroid-blocking immunoglobulins in congenital hypothyroidism. *Journal of Clinical Endocrinology and Metabolism*.

[36] Pacaud D., Huot C., Gattereau A. (1995). Outcome in three siblings with antibody-mediated transient congenital hypothyroidism. *The Journal of Pediatrics*.

[37] Evans C., Gregory J. W., Barton J. (2011). Transient congenital hypothyroidism due to thyroid-stimulating hormone receptor blocking antibodies: a case series. *Annals of Clinical Biochemistry: International Journal of Laboratory Medicine*.

